# Transcranial direct current stimulation to the left dorsolateral prefrontal cortex enhances early dexterity skills with the left non-dominant hand: a randomized controlled trial

**DOI:** 10.1186/s12967-023-03989-9

**Published:** 2023-02-24

**Authors:** Akihiro Watanabe, Daisuke Sawamura, Hisato Nakazono, Yukina Tokikuni, Hiroshi Miura, Kazuhiro Sugawara, Kanako Fuyama, Harukazu Tohyama, Susumu Yoshida, Shinya Sakai

**Affiliations:** 1 Graduate School of Health Sciences, Hokkaido University, Sapporo, 060-0812 Japan; 2 Department of Rehabilitation Science, Faculty of Health Sciences, Hokkaido University, Sapporo, Hokkaido 060-0812 Japan; 3 Department of Occupational Therapy, Faculty of Medical Science, Fukuoka International University of Health and Welfare, Fukuoka, 814-0001 Japan; 4 Department of Physical Therapy, Sapporo Medical University, Sapporo, 060-8556 Japan; 5 Data Science Center, Promotion Unit, Institute of Health Science Innovation for Medical Care, Hokkaido University Hospital, Sapporo, 060-8648 Japan; 6 Department of Rehabilitation Sciences, Health Sciences University of Hokkaido, Tobetsu, 061-0293 Japan

**Keywords:** Transcranial direct current stimulation, Motor learning, Dorsolateral prefrontal cortex, Non-dominant hand, Fine motor, Cognitive aspects

## Abstract

**Background:**

The left dorsolateral prefrontal cortex (DLPFC) is involved in early-phase manual dexterity skill acquisition when cognitive control processes, such as integration and complexity demands, are required. However, the effectiveness of left DLPFC transcranial direct current stimulation (tDCS) on early-phase motor learning and whether its effectiveness depends on the cognitive demand of the target task are unclear. This study aimed to investigate whether tDCS over the left DLPFC improves non-dominant hand dexterity performance and determine if its efficacy depends on the cognitive demand of the target task.

**Methods:**

In this randomized, double-blind, sham-controlled trial, 70 healthy, right-handed, young adult participants were recruited. They were randomly allocated to the active tDCS (2 mA for 20 min) or sham groups and repeatedly performed the Purdue Pegboard Test (PPT) left-handed peg task and left-handed assembly task three times: pre-tDCS, during tDCS, and post tDCS.

**Results:**

The final sample comprised 66 healthy young adults (mean age, 22.73 ± 1.57 years). There were significant interactions between group and time in both PPT tasks, indicating significantly higher performance of those in the active tDCS group than those in the sham group post tDCS (p < 0.001). Moreover, a greater benefit was observed in the left-handed assembly task performance than in the peg task performance (p < 0.001). No significant correlation between baseline performance and benefits from tDCS was observed in either task.

**Conclusions:**

These results demonstrated that prefrontal tDCS significantly improved early-phase manual dexterity skill acquisition, and its benefits were greater for the task with high cognitive demands. These findings contribute to a deeper understanding of the underlying neurophysiological mechanisms of the left DLPFC in the modulation of early-phase dexterity skill acquisition.

*Trial registration*: This study was registered in the University Hospital Medical Information Network Clinical Trial Registry in Japan (UMIN000046868), *Registered February 8, 2022 *https://center6.umin.ac.jp/cgi-open-bin/ctr_e/ctr_view.cgi?recptno=R000053467

**Supplementary Information:**

The online version contains supplementary material available at 10.1186/s12967-023-03989-9.

## Background

Motor skill learning is crucial for optimizing human behavior and essential in a variety of daily life situations. The motor learning theory of Fitts and Posner [1] presents a sequential three-stage model of motor learning—cognitive, associative, and autonomous—that requires increasingly less attention and working memory as motor skills improve. The early phase of motor skill learning corresponds to the cognitive stage and is characterized by inconsistent and inefficient movement and high cognitive demand for motor control. Previous neuroimaging studies have provided evidence that early-phase skill acquisition involves the bilateral dorsolateral prefrontal cortex (DLPFC) as well as cortical motor areas such as the primary motor cortex, premotor cortex, and supplementary motor area [2–6]. The DLPFC is involved in top-down attention, working memory, and executive functions such as planning and monitoring [7–13]. DLPFC activity has recently been recognized as the most critical neural bases underlying cognitive processing in early-phase motor learning [14, 15], as it is involved in cognitive control of sensory input, future action planning [3, 16], and encoding and maintenance of declarative memory [4, 17].

Moreover, the DLPFC is highly activated during the performance of unfamiliar or complex fine motor skill tasks with bimanual and unimanual non-dominant hands [5, 6, 18, 19]. Specifically, the left DLPFC has also been reported to be a key brain region involved in tool use or object operation behaviors [5, 20, 21], and this left-lateralized activation is independent of handedness [20, 22, 23].

Transcranial direct current electrical stimulation (tDCS) is a noninvasive brain stimulation technique that can modulate neuronal excitability in cortical and subcortical areas by inducing polarity-specific membrane potential changes. Depending on stimulus intensity and duration, anodal tDCS stimulation has generally been shown to increase neuronal excitability and to promote spontaneous neuronal firing and long-term potentiation, whereas cathodal stimulation has been shown to induce the opposite effect [24–26]. However, the effects of anodal tDCS on motor and cognitive performance reportedly differ depending on the targeted brain region and task type [27, 28]. To resolve this controversy, recent studies of tDCS on motor learning highlight the importance of employing standardized, prospective, pre-registered, hypothesis-driven studies to improve transparency, reproducibility, and consistency [29].

Most previous studies evaluating tDCS effects in upper-limb motor learning in healthy individuals targeted the cortical motor areas and cerebellum [30]. These studies demonstrated that anodal tDCS facilitates learning of many different tasks: the implicit sequence learning task [31]; explicit sequence learning task [32]; and visuomotor learning tasks such as the drawing [33], pegboard [34], and pinch force [35] tasks. However, little is known about the neural bases of DLPFC activity in the early stages of upper-limb motor learning. Thus, Ashcroft et al. [36] applied 2-mA active or sham tDCS for 15 min on the left DLPFC of 40 young, healthy, medical students performing a surgical knot-tying task after 1-h of training. Compared to the sham condition, the participants demonstrated significantly improved performance and reduced subjective workload during and post tDCS. Nakashima et al. [31] also applied 2-mA anodal tDCS on the left DLPFC for 20 min to 16 healthy, young adults to test learning gain in the serial reaction time task using a within-participants cross-over design, and demonstrated a significantly decreased reaction time. Vergallito et al. [37] delivered a 1.5-mA current for 20 min on the left and right DLPFC to 24 young adults and found a significantly higher accuracy on the paced finger-tapping task with high difficulty level for left stimulation compared to either right stimulation or sham conditions.

Commonly, these studies targeting the DLPFC demonstrated significantly improved performance in the active tDCS group compared to the sham group; meanwhile, the results of most previous studies targeting the other cortical motor areas and cerebellum were extremely inconsistent [30, 32–35]. Additionally, these results suggest that matching the related brain region according to the stage of motor skill learning is important to improve the effectiveness of tDCS. Furthermore, these findings suggest that stimulation of the left DLPFC at 1.5–2.0 mA for 15–20 min is effective for upper-limb motor learning, which requires cognitive processing, and that tasks with higher cognitive demand produce larger learning gains. However, the effect of tDCS on the left DLPFC during the early phase of motor learning is only partially understood, and its impact on learning unfamiliar tasks with the non-dominant hand is unknown. Moreover, it remains unclear whether its effectiveness depends on the cognitive demand of the task. Therefore, we focused on the involved brain region and its assumed functions according to the motor learning stage and aimed to evaluate whether tDCS of the left DLPFC, which is highly activated via manipulation of objects, improves early-phase motor learning by the non-dominant hand and if the efficacy depends on the cognitive demand of the target task. We selected the Purdue Pegboard Test (PPT), comprising a simple peg task and an assembly task, because it allowed us to assess left-lateralized DLPFC activity and classify the degree of cognitive demand according to presence or absence of movement sequences in the task. Motor sequence learning, which is part of the assembly task, is a process that involves independent movements, eventually leading to a multi-element sequence that can be performed quickly and accurately [38, 39], and requires cognitive processes such as attention and working memory [40–42]. Moreover, previous studies reported the dissociation of learning effect between the sequential order of a movement sequence and its motor control components [43–46]. These two components are learned in parallel, with different time courses of learning effect, and it is thought that in the early phase of motor learning, improvements are chiefly dominated by learning the sequential characteristics of the movements, which require cognitive load [44, 47].

Therefore, we hypothesized that anodal tDCS on the left DLPFC might enhance early manual dexterity skill acquisition of the non-dominant hand and would lead to greater learning gain in tasks with high cognitive demand.

## Methods

### Trial design and participants

This study was a double-blinded, randomized, sham-controlled trial with stratification by age, sex, and handedness score (Edinburgh Handedness Inventory, [EHI]) [48]. Seventy healthy, right-handed, young adults were recruited within 3 months (March 2022 through May 2022), based on the following inclusion and exclusion criteria. The inclusion criteria were (1) aged 20–30 years and (2) no experience performing the PPT with their non-dominant left hand. The exclusion criteria were (1) history of neurological or psychiatric disorders, (2) functional limitations of both or either upper limb affecting task performance, (3) insufficient safety for transcranial electrical stimulation evaluated by a safety questionnaire (metal implants, pacemaker, history of epilepsy, and pregnancy [49]), and (4) a score of < 70 points on the EHI. The required sample size for this study was calculated a priori by power analysis with G power 3.1 [50] based on the results of a previous study [51]. The sample size for achieving a 0.95 statistical power at a significance level of α = 0.05 and given effect size f = 0.226 for a time × group interaction using a linear mixed effect modeling was n = 58. To ensure a conservative estimation, 20% was added considering the possibility of dropouts or outliers, for a final sample size of n = 70. Independent investigators randomly allocated all participants into the active tDCS (n = 35; 17 women) or sham (n = 35; 18 women) group according to a computer-generated stratified blocked randomization list with stratification factors (age, sex, and EHI scores). The block size for the randomization was randomly chosen for each block as four to six participants, with a 1:1 allocation. Both the participants and other investigators except for the investigators with the role of random allocation were blinded to brain stimulation assignments.

Written informed consent was obtained from all volunteers along with demographic information, handedness, and their experience with the PPT.

This study was approved by the relevant ethics committee (Approval Number: 21–85) and registered in the University Hospital Medical Information Network Clinical Trial Registry in Japan (UMIN000046868). Additionally, this study conformed to Consolidated Standards of Reporting Trials (CONSORT) guidelines. All experiments were conducted in accordance with the Declaration of Helsinki.

### Experimental procedure

**Fig. 1:**
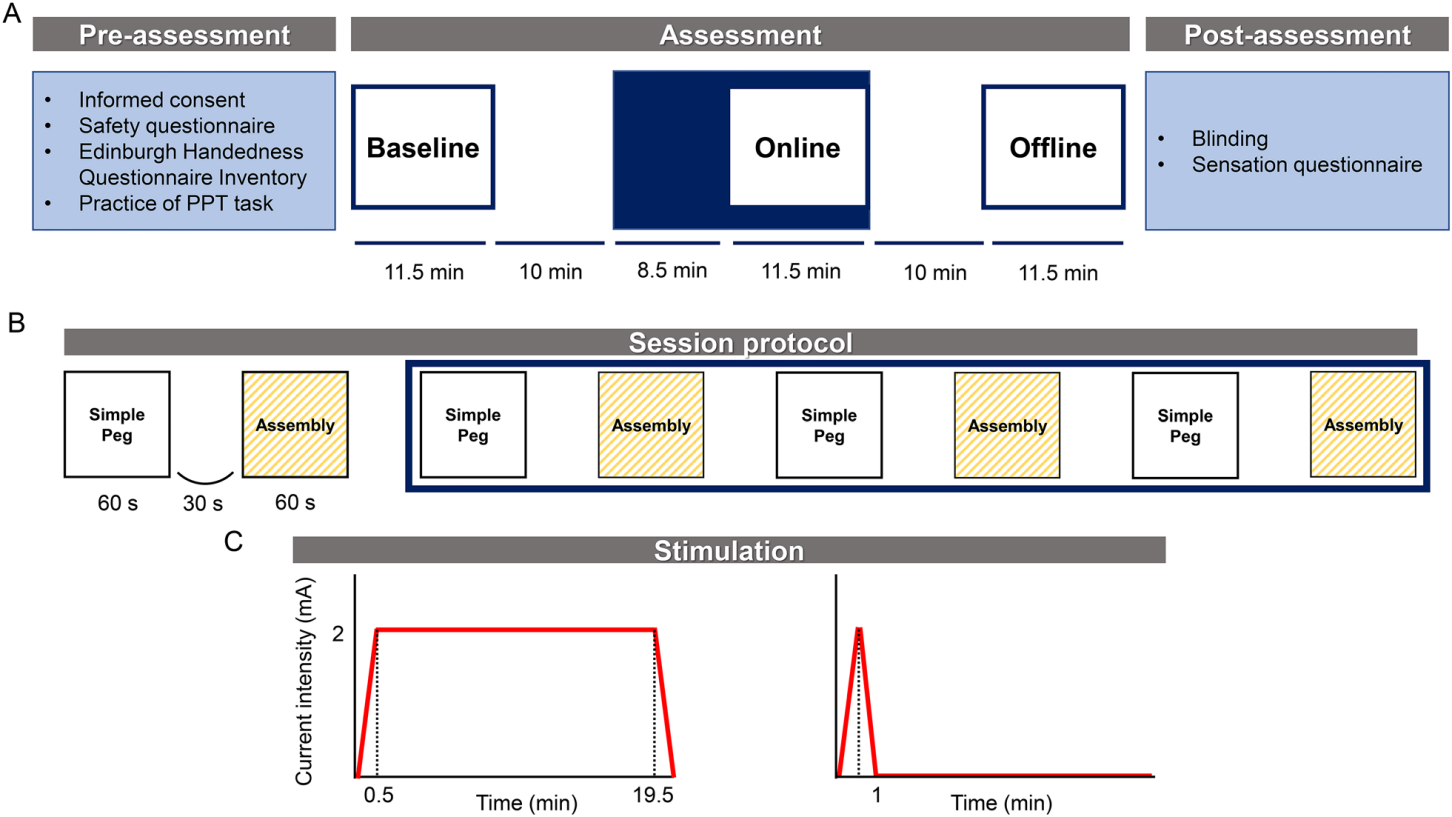
Schematic representation of the experimental procedure and design. **A** Time course of the study design. This study consisted of the following three phases: Pre-assessment, Assessment, and Post-assessment. The assessment phase consisted of three sessions: baseline, online, and offline. Navy blue shows the 20-min tDCS stimulation period. **B** The protocol in each of the three assessment sessions consisted of four repetitions of the two subtests of the PPT, the left-handed peg task and left-handed assembly task. Data from the first trial was removed from analysis, considering the possibility of generating large variation in task performance. **C** Schematic illustration of active and sham stimulation. The left figure shows active stimulation, a sustained 2-mA current delivered for 20 min. The right figure shows sham stimulation, a ramp up to 2 mA followed by an immediate ramp down. *PPT* Purdue Pegboard Test, *tDCS* transcranial direct current stimulation

The experimental procedure consisted of three phases: pre-assessment, assessment, and post-assessment (Fig. 1A), all of which were completed in a single day. During the pre-assessment phase, all recruited participants were requested to answer the two questionnaires (safety questionnaire for transcranial electrical stimulation and EHI) first. Thus, only the participants who managed to ensure safety and to reach the criterion score of the EHI were selected. Subsequently, the participants were informed about the course of the experiment and asked to practice the two subtests of the PPT 1 time each.

In the assessment phase, all participants performed the two PPT subtests (the left-handed peg and the assembly task, see the Two subtests of PPT subsection for a detailed description) at three assessment periods: baseline, during tDCS (online), and post tDCS (offline) (Fig. 1B). Either sham or anodal tDCS stimulation targeting the left DLPFC was used. The participants performed each subtest 4 times, with a 60-s task performance time and 30-s inter-task intervals, for a total duration of 11.5 min. For the initial 8.5 min, participants did not perform the task and received tDCS stimulation alone. To ensure thorough blinding in the analyzing investigators, each independent investigator assessed the PPT and delivered the stimuli. Other experimenters were responsible for the pre- and post-assessment procedures.

A 10-min rest period separated each assessment phase. Participants were informed that they would receive “two different intensities of forehead stimulation” and were blinded to the stimulation mode. A schematic illustration of active and sham stimulation is shown in Fig. 1C.

After stimulation, during the post-assessment phase, the success of blinding was measured by asking participants to guess whether they had received active or sham tDCS to justify its effectiveness on early-phase dexterity acquisition. Additionally, subjective discomfort (pain) was measured using a sensation questionnaire to identify participants’ adverse effects, safety, and tolerability [52]. Based on the time course of previous experiments focusing on the early phase of motor learning [31, 36, 51], all three assessment phases in this study were defined as early phase of learning assessments.

### Two subtests of PPT

The PPT is widely used as a hand function test in therapy, rehabilitation, and treatment to evaluate dexterity performance. It assesses dexterity of precision grip using two subtests [53]. In the peg task, the participant inserts a peg into a hole in a board with either hand, and in the assembly task, a collar and two washers are sequentially inserted onto the peg with both hands. In this study, the assembly task was performed only with the non-dominant left hand instead of both hands.

The left-handed peg task required participants to insert as many pegs into the holes as possible within 60 s, and the score is the number of pegs inserted correctly. In the assembly task, participants were required to use their left hand to insert the peg and add two washers and a collar in a certain order within 60 s. Compared to the simple peg task, higher cognitive demand is required to sequentially assemble four parts with different shapes.

The score of the assembly operation task is the total number of parts from the completed assemblies and uncompleted assemblies. Each of these tasks was repeatedly conducted 4 times in each assessment session. The data in the first trial was discarded from the analysis in consideration of the possibility of a large variation in task performance and greater improvement from the first trial to second trial (Fig. 1B). We ensured participants received no guidance and feedback while performing the task to assess the net impact of tDCS.

### Transcranial direct current stimulation

**Fig. 2:**
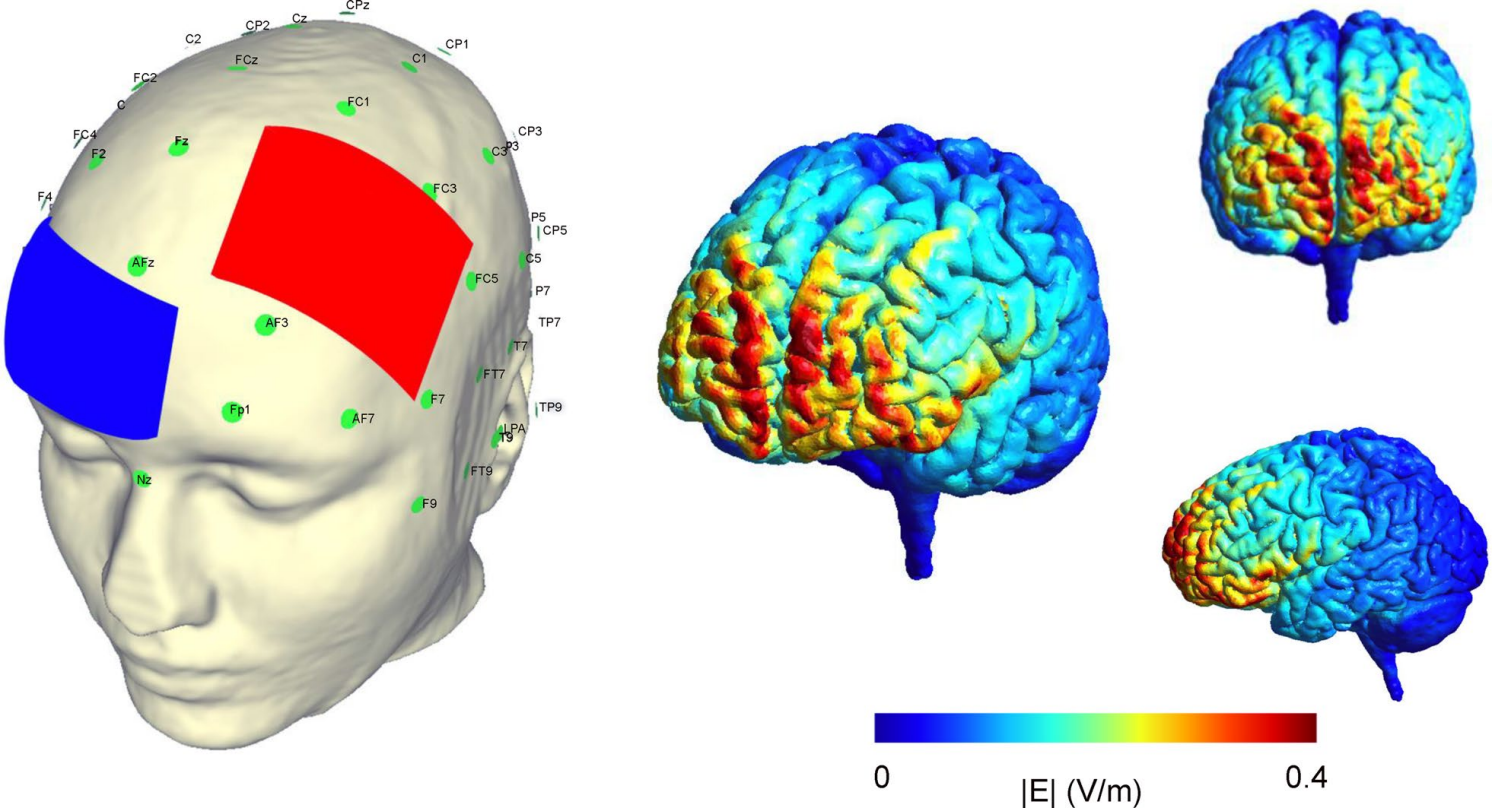
Left DLPFC (F3) and orbitofrontal cortex (Fp2) electrode placement (international 10/20 system). The left image shows the electrode configurations with the anode (red) over F3 and cathode (blue) over Fp2. The right image shows the underlying cortical electric field on different directions of the brain map. The horizontal color bar indicates the electric field magnitude expressed in norm E (V/m). *DLPFC* dorsolateral prefrontal cortex

Stimulation was delivered using a DC-STIMULATOR PLUS (NeuroConn GmbH, Ilmenau, Germany) through a pair of 0.9% saline-soaked 5 × 7 cm electrodes, resulting in a current density of 0.057 mA/cm^2^, which was well within the current safety standards [54]. For both active and sham stimulation, a pair of electrodes was placed over the left DLPFC (F3) and right supraorbital cortex (Fp2) as anode and cathode, according to the international 10–20 EEG system, affixed by two circumferential straps (Fig. 2). The allocation of electrodes was determined based on prior studies demonstrating improved cognitive and behavioral measures [55, 56]. Hill et al. [55] suggested that higher-current and longer-duration tDCS stimulation to the DLPFC is more effective at modulating cognitive function in a healthy population. Furthermore, previous studies demonstrated that anodal 2-mA tDCS for 20 min was suitable to increase cortical excitability and produce long-lasting effects [57, 58]. Accordingly, we adopted this paradigm. Figure 2 shows a computational simulation model [59] of the electrode placement and underlying cortical electric field. The electrode placement and the electric field simulation were visualized using SimNIBS (Version 3.2). In the active tDCS group, the stimulation protocol consisted of a 30 s gradual ramping up to 2-mA for the duration of the 20 min online session, followed by a 30 s gradual ramping down. In the sham group, sham stimulation consisted of 60 s of 2 mA stimulation administered at the beginning of the 20 min stimulation delivered at the beginning of the 20 min (30 s ramp-up and ramp-down) and 0 mA for the remainder of the period.

### Statistical analyses

Baseline characteristics and the assessment of blinding and sensation questionnaire were analyzed using a t-test or chi-square test.

For the number of completions in each PPT subtest, a 2 × 3 mixed-design analysis of variance (ANOVA), with group (active tDCS or sham) as the between-participants factor and time (baseline, online, and offline sessions) as the within- participant factor and effect sizes calculated as partial eta squared (*η*^*2*^_*p*_), was performed after confirming normality using the Shapiro–Wilk test. The Greenhouse–Geisser correction was applied for an ANOVA to correct degrees of freedom when the assumption of sphericity was violated. The Bonferroni method was used for multiple comparisons in each PPT subtest (the corrected P was calculated by multiplying the P value by 3 in each post-hoc test in two factors, respectively). In addition, the number of completions in each PPT subtest was transformed into standardized Z-scores (mean = 0.0, standard deviation = 1.0) based on the baseline values in each group. The effectiveness of left-prefrontal tDCS depending on cognitive demand of the target task was tested using a 2 × 2 × 2 mixed-design ANOVA with group (active tDCS or sham) as the between-participants factor and time (online and offline sessions) and task type (simple peg and assembly tasks) as the within-participant factors. Finally, correlation analyses were performed to examine the relationship between the PPT task performance at the baseline with that in each evaluation period (offline performance – baseline performance and online performance – baseline performance) in the active tDCS group using Pearson’s product-moment correlation analysis. All statistical analyses were performed using SPSS (version 25.0; IBM Corp., Armonk, NY, USA), and the α level was set at 0.05.

## Results

### Baseline characteristics and the assessment after stimuli

**Fig. 3:**
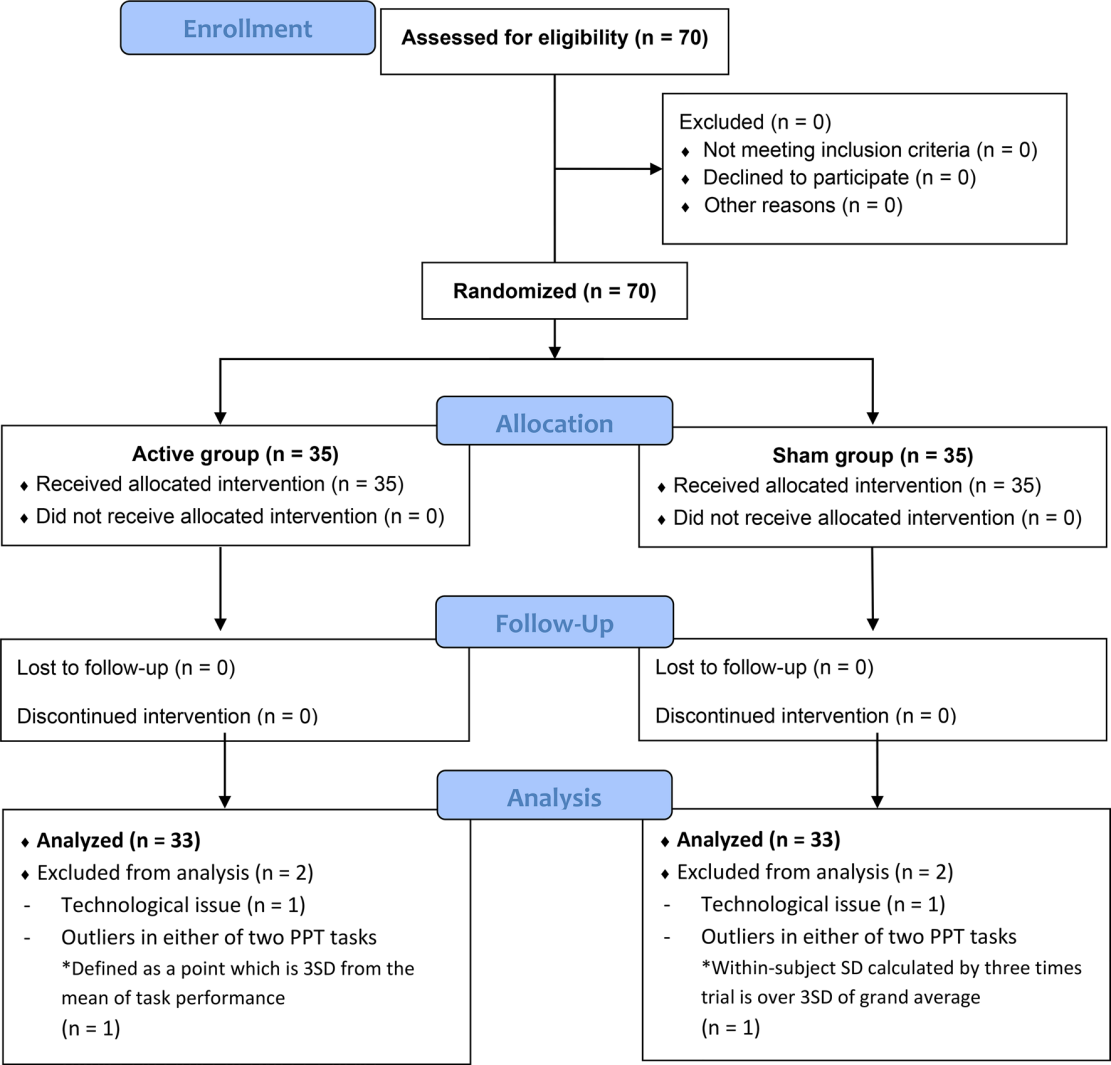
CONSORT flow diagram. Flow diagram summarizing the steps and number of participants excluded with implementation of each eligibility criteria culminating in the final data analysis. *PPT* Purdue Pegboard Test, *SD* standard deviation

Figure 3 shows the flow diagram for the procedure to final analysis. The final sample comprised 66 healthy, right-handed, young adults (aged 20–27 years; mean age, 22.73 ± 1.57; mean EHI score, 97.41 ± 6.52). Table 1 shows the baseline characteristics and the assessment of blinding and perception of stimulation in each group.

**Table 1:** Baseline characteristics and assessment of blinding success and sensation

	Active (n = 33)	Sham (n = 33)	p-value	Statistics
Participant demographics				
Age (years; mean [SD])	22.7 (1.33)	22.8 (2.07)	0.83	t = − 0.21
Sex			0.62	χ^2^ = 0.24
Male (n (%))	17 (51.5)	15 (45.5)		
Female (n (%))	16 (48.5)	18 (54.5)		
Edinburgh Handedness Inventory mean (SD)	97.52 (6.80)	97.91 (5.53)	0.80	t = − 0.26
Blinding			0.22	χ^2^ = 1.54
Felt stimulated (n (%))	17 (51.5)	12 (36.3)		
Felt non-stimulated (n (%))	16 (48.5)	21 (63.7)		
Sensation questionnaire (Median [IQR])				
Itching	1.0 (0.0–1.0)	1.0 (0.0–1.0)	0.30	χ^2^ = 2.43
Pain	0.0 (0.0–0.0)	0.0 (0.0–0.5)	0.56	χ^2^ = 1.15
Burning	0.0 (0.0–0.0)	0.0 (0.0–0.0)	0.39	χ^2^ = 0.73
Warmth/Heat	0.0 (0.0–0.5)	0.0 (0.0–0.0)	0.55	χ^2^ = 0.36
Pinching	0.0	0.0	N/A	N/A
Metallic	0.0	0.0	N/A	N/A
Fatigue	0.0 (0.0–0.5)	0.0 (0.0–1.0)	0.73	χ^2^ = 0.64
Others	0.0 (0.0–0.0)	0.0 (0.0–0.0)	0.54	χ^2^ = 1.22

*SD* standard deviation, *IQR* interquartile range

There were no significant differences in age, sex, or EHI score between the training and control groups. In addition, no significant differences in the blinding success and perception of stimulation between the two groups were found (Table 1).

### PPT tasks performance

**Fig. 4:**
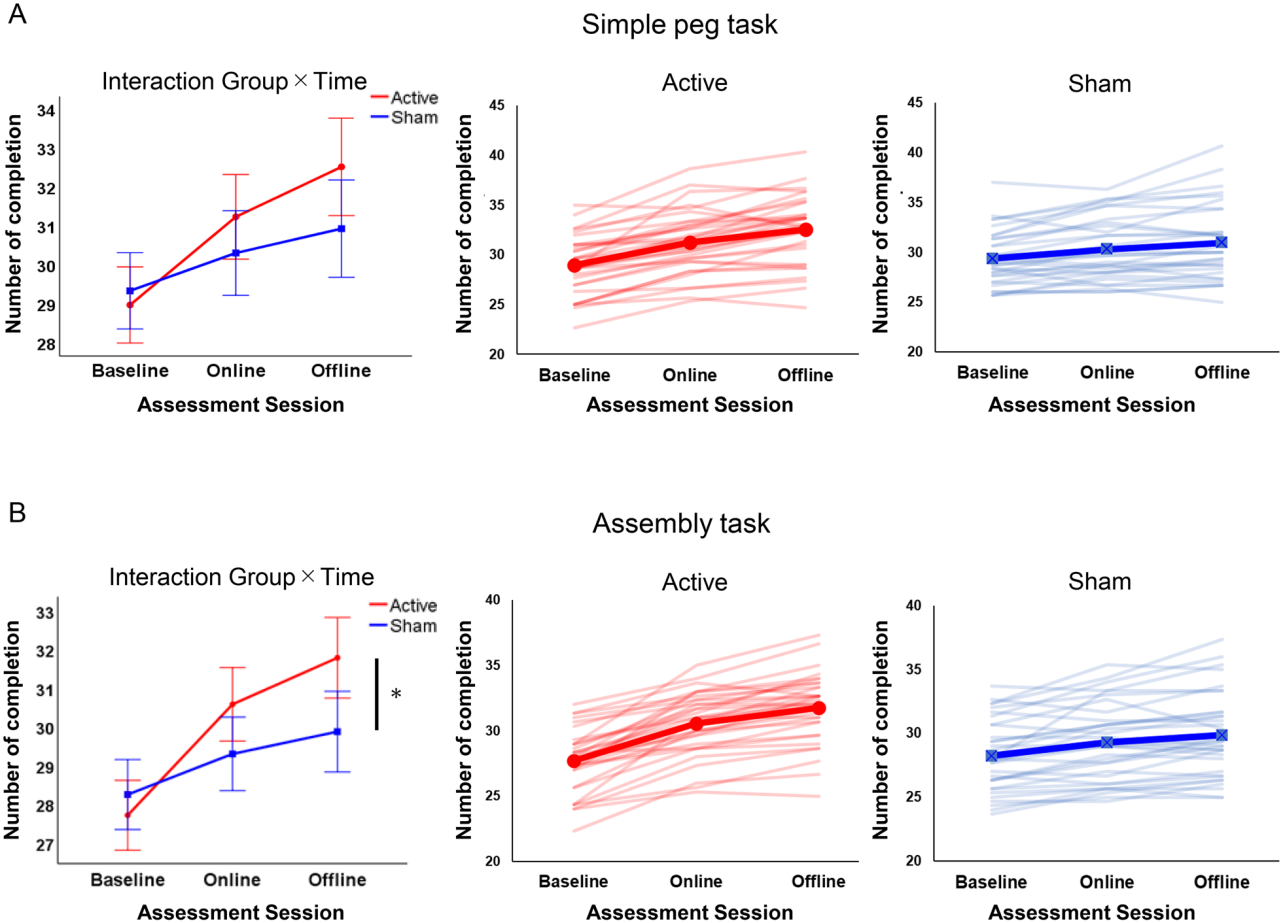
Effects of tDCS stimulation on the number of completions for the two PPT subtests. **A** The results of the number of completions in the simple peg task. The left panel shows the results of 2 × 3 mixed-design analysis of variance for the left-handed simple peg task. The middle and right panels show the individual performance of the simple peg task across the three assessment sessions in the active and sham groups, respectively. The bold lines show the mean number of completions in each group performance. **B** The results of the number of completions in the assembly task. The left panel shows the results of 2 × 3 mixed-design analysis of variance for the left-handed assembly task. The middle and right panels show the individual performance of the assembly task across the three assessment sessions in the active and sham groups, respectively. The bold lines show the mean number of completions in each group performance. Error bars indicate the standard error of the mean. * = Corrected p < 0.05. *PPT* Purdue Pegboard Test, *tDCS* transcranial direct current stimulation

Table 2 shows the average performance of each PPT subtest at all assessment sessions in both groups. In the left-handed peg task, a 2 × 3 mixed-design ANOVA revealed a significant main effect of time (F[2,128] = 101.44, p < 0.001, *η*^*2*^_*p*_ = 0.162) and a significant time × group interaction (F[2,128] = 14.78, p < 0.001, *η*^*2*^_*p*_ = 0.188) but no significant main effect of group (F[1, 64] = 0.90, p = 0.346, *η*^*2*^_*p*_ = 0.014) (Fig. 4A). A post-hoc t-test with time showed significant differences among all three evaluation periods in both groups (Offline > Online > Baseline, all corrected p < 0.001) (Table 3), indicating gradual improvement of task performance over the 3 time periods. Regarding group comparison, a post-hoc t-test with group showed no significant differences in all evaluation periods (All corrected p ≥ 0.23).

**Table 2:** Average performance of PPT tasks

	Active (n = 33)			Sham (n = 33)		
	Baseline	Online	Offline	Baseline	Online	Offline
Peg task						
Number of completions	28.98 (2.90)	31.24 (3.17)	32.53 (3.44)	29.34 (2.72)	30.31 (3.07)	30.94 (3.73)
Z-scores	–	0.79 (1.11)	1.24 (1.21)	–	0.36 (1.15)	0.59 (1.39)
Assembly task						
Number of completions	27.70 (2.38)	30.57 (2.39)	31.77 (2.66)	28.23 (2.79)	29.28 (3.03)	29.86 (3.29)
Z-scores	–	1.22 (1.02)	1.74 (1.14)	–	0.38 (1.10)	0.59 (1.20)

All data are shown as means (standard deviations)

*PPT* Purdue Pegboard Test

**Table 3:** Results of the post-hoc test and Cohen’s d values for two PPT tasks

	Statistics	Active (n = 33)		Sham (n = 33)			Statistics, 95% CI, Cohen’s d(Active vs. Sham)		
		95% CI (mean difference)	Cohen’s d	Statistics	95% CI	Cohen’s d	Baseline	Online	Offline
Simple peg task									
Baseline vs. Online	t = 8.63 p < 0.001^a^	1.73–2.80	0.75	t = 4.39 p < 0.001^a^	0.52–1.42	0.34	t = -0.53 p = 1.00^a^, − 1.75–1.02, Cohen’s d = 0.13	t = 1.21 p = 0.65^a^, − 0.60–2.46, Cohen’s d = 0.30	t = 1.80 p = 0.23^a^, − 0.18–3.35, Cohen’s d = 0.45
Baseline vs. Offline	t = 10.95 p < 0.001^a^	2.87–4.20	1.12	t = 6.45 p < 0.001^a^	1.09–2.10	0.50			
Online vs. Offline	t = 5.00 p < 0.001^a^	0.76–1.80	0.39	t = 2.79 p = 0.026^a^	0.17–1.08	0.18			
Assembly task									
Baseline vs. Online	t = 10.89 p < 0.001^a^	2.33–3.41	1.22	t = 4.56 p < 0.001^a^	0.58–2.15	0.37	t = − 0.84 p = 1.00^a^, − 1.81–0.74, Cohen’s d = 0.21	t = 1.91 p = 0.18^a^, − 0.60–2.63, Cohen’s d = 0.48	t = 2.59 p = 0.036^a^, 0.44–3.38, Cohen’s d = 0.65
Baseline vs. Offline	t = 13.41 p < 0.001^a^	3.45–4.69	1.64	t = 6.32 p < 0.001^a^	1.10–2.15	0.54			
Online vs. Offline	t = 7.08 p < 0.001^a^	0.86–1.55	0.48	t = 3.02 p = 0.015^a^	0.19–0.96	0.19			

^a^All p values were Bonferroni corrected

*PPT* Purdue Pegboard Test, *CI* confidence interval

Regarding the left-handed assembly task, a 2 × 3 mixed-design ANOVA revealed a significant main effect of time (F[1.64,104.85] = 147.28, p < 0.001, *η*^*2*^_*p*_ =  0.697) and a significant time × group interaction (F[1.64,104.85] = 27.947, p < 0.001, *η*^*2*^_*p*_ = 0.304) but no significant main effect of group (F[1, 64] = 1.826, p = 0.181, *η*^*2*^_*p*_ = 0.028) (Fig. 4B). A post-hoc t-test showed significant differences among all time periods in both groups (Offline > Online > Baseline, all corrected p < 0.001) (Table 3), indicating gradual improvement of task performance over time. Additionally, a post-hoc t-test only showed significantly higher performance in the active tDCS group compared to the control group in the offline session (t[32] = − 2.59, corrected p = 0.036, 95% confidence interval [CI] for the mean difference: 0.44–3.38, Cohen’s d = 0.65) but no difference in the baseline and online sessions (corrected p = 1.00 and 0.18, respectively).

### Comparison of normalized PPT task performance

**Fig. 5:**
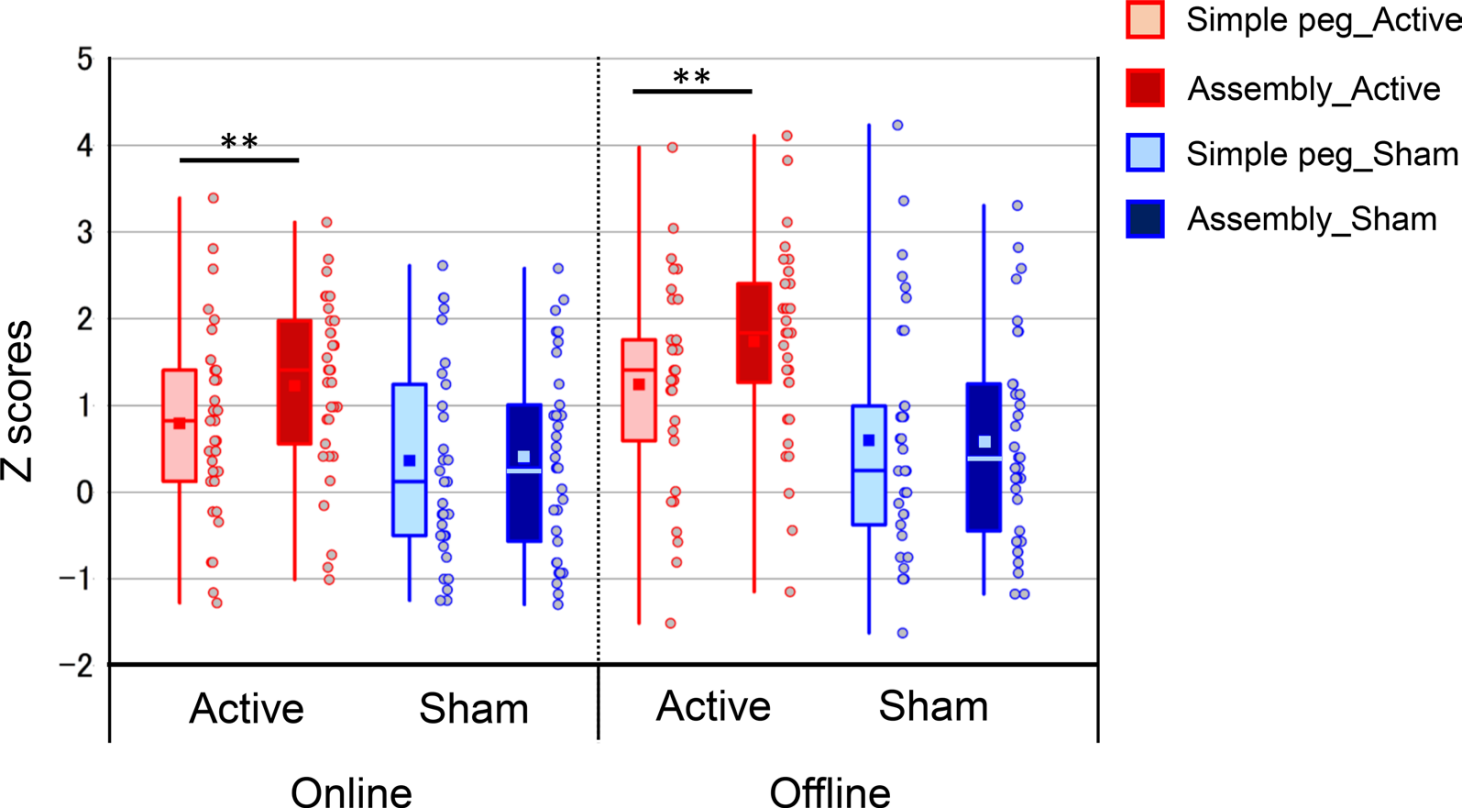
Effects of tDCS stimulation on the normalized Z-scores of the two PPT subtests. The results of 2 × 2 × 2 mixed-design analysis of variance of the normalized Z-scores with group as the between-participant factor and time and task type as the within-participant factors. The displayed points show the individual Z-scores. Error bars indicate the 95% confidence interval; the bottom and top of each box, the 25th and 75th percentiles; and the line and square inside the box, the 50th percentile (median) and the mean, respectively. ** = p < 0.001. *PPT* Purdue Pegboard Test, *tDCS* transcranial direct current stimulation

Table 2 shows the normalized Z-scores of each PPT subtest in online and offline sessions in both groups. A 2 × 2 × 2 mixed-design ANOVA with group as the between-participants factor and time and task type as the within-participant factors revealed a significant main effect of all factors (group: F[1, 64] = 7.97, p = 0.006, *η*^*2*^_*p*_ = 0.111; time: F[1, 64] = 60.59, p < 0.001, *η*^*2*^_*p*_ = 0.486; task type: F[1, 64] = 60.59, p = 0.004, *η*^*2*^_*p*_ = 0.124) and significant time × group interaction (F[1, 64] = 8.25, p = 0.006, *η*^*2*^_*p*_ = 0.114) and task type × group interaction (F[1, 64] = 9.07, p = 0.005, *η*^*2*^_*p*_ = 0.118, (see the table in Additional File 1: Table S1). The active tDCS group showed a significantly higher value in the left-handed assembly task compared to the left-handed peg task in both online and offline sessions (t[32] = − 3.90, p < 0.001, 95% CI: 0.20–0.66, Cohen’s d = 0.41; t[32] = − 4.62, p < 0.001, 95% CI 0.28–0.72, Cohen’s d = 0.42, respectively) (Fig. 5). In the sham group, no significant difference was observed in either the online or offline session (online: t[32] = 0.168, p = 0.868, 95% CI − 0.21 to 0.25, Cohen’s d = 0.02; offline: t[32] = − 0.04, p = 0.969, 95% CI − 0.30 to 0.29, Cohen’s d = 0.004).

### Relationship between the PPT task performance at baseline and changes in the PPT task performance in each evaluation period

**Fig. 6:**
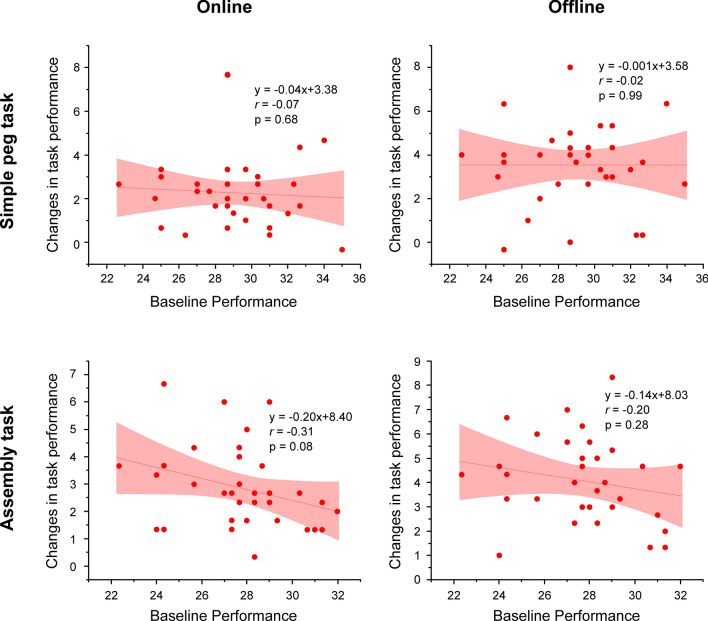
Scatterplots for relationship between PPT task performance at baseline and online and offline session changes. Left, scatterplots of each of the two tasks (simple peg task and assembly task) in the online session. Right, scatterplots of each of the two tasks in offline assessment. The straight and curved lines indicate the mean and 95% confidence interval, respectively. *PPT* Purdue Pegboard Test

There was no significant correlation between the PPT task performance at baseline and changes in each task performance in online and offline sessions in the active tDCS groups (all, p > 0.08) (Fig. 6).

## Discussion

This prospective, hypothesis-driven, double-blind, randomized controlled trial demonstrated that tDCS over the left DLPFC significantly enhanced early-phase upper-limb motor learning in both the left-handed simple peg and assembly tasks, which was the result anticipated by our hypothesis. Importantly, this study fulfilled the suggested criteria in a recent consensus and critical position article on tDCS [28] by enhancing transparency, reproducibility, and standardization. In this study, using the PPT, we tested the impact of tDCS on the left DLPFC and found that it improved early-phase manual dexterity skills with the non-dominant hand, and its effectiveness depended on the cognitive demand of the target task. In these two tasks with different cognitive demands, significant main effects of time, indicating gradual improvement throughout the experiment, were observed. This is the characteristic learning effect from repetition of performing tasks in the initial stage. However, regardless of this effect, a significant interaction of group × time (Fig. 4), indicating improvement of task performance in the active tDCS group during the offline session, was found for both tasks. These results suggest that tDCS was effective in promoting spontaneous neuronal firing and long-term potentiation in the left DLPFC, resulting in improved learning of unfamiliar operations requiring non-dominant hand movement. Regarding blood oxygenated level-dependent activity (BOLD) or regional cerebral blood flow (rCBF), given that anodal tDCS targeting the DLPFC has been shown to induce increased BOLD signal and rCBF in the cortical and subcortical regions [60, 61], it may be postulated that in this study, tDCS also contributed to the upregulation of DLPFC activity. Moreover, these findings support those of previous reports targeting the left DLPFC [31, 36, 37] and contribute to establishing further evidence for tDCS on the left DLPFC in the early stages of upper-limb motor learning. Furthermore, these results suggest that matching the involved brain region and its assumed functions according to the motor learning stage is critical for successful implementation of tDCS, enabling it to exert its effects in motor learning. In support of this, a study reported that monkeys showed a deficit in new skill learning after DLPFC lesions [62].

In the offline assessment, the post-hoc test revealed that active tDCS significantly improved learning in the assembly task but did not have an effect in the simple peg task. A meta-analysis reported that tDCS over the DLPFC significantly improved offline working memory performance in healthy cohorts; however, no effect was observed in online performance [55]. The online effects of tDCS depend on neurobiological processes taking place during the event, as opposed to synaptic-driven changes (i.e., modulation of GABAergic activity) occurring after the stimulation, which are considered to drive the offline effects and influence behavioral responses more strongly [63, 64]. Our results are in agreement with the idea of synaptic changes having a larger impact on behavioral responses than mere changes in membrane potential, as previously suggested [55]. Previous studies targeting the left DLPFC reported that the effect of 20 min of anodal tDCS lasted up to 40 min [65, 66]. Although we only demonstrated short-lasting effects, 10 min after stimulation, longer lasting effects may be expected. Thus, further study is needed to examine the longer long-lasting effect of tDCS.

Regarding the effect size, our results of two PPT tasks were far above the size reported for anodal tDCS on the primary motor cortex comparing sham tDCS reported in a previous meta-analysis in upper limb dexterity motor learning (0.04 (95% CI 0.01–0.07) [30] at online and offline assessments. Furthermore, similar results were reported when the comparison was limited to the PPT task (0.07 and 0.05, respectively) [34, 67]. Alternatively, in a recent report the effect size observed when the left DLPFC was targeted on implicit motor learning to acquire new motor skill was greater than that observed in previous reports targeting the primary motor cortex (effect size in post tDCS assessment: 0.55) [31]. This effect size is comparable to that observed in the simple peg task at offline assessment in our study, as well as in the assembly task at both online and offline assessments. Hence, our results suggest that anodal tDCS stimulation of the DLPFC may be beneficial for promoting early motor learning in the upper limb, especially in the left hand. Interestingly, the effect size of time in the active tDCS group was almost constantly magnified compared with that in the sham tDCS group across whole assessments in each of the two tasks; further, the effect sizes of time in the active tDCS group were nearly two and 3 times as large as those in the sham tDCS group at all assessment sessions in the simple peg and assembly tasks, respectively. The results suggest a greater likelihood to establish a close linear relationship between the simple learning effect in non-dominant left-handed performance of each task and learning gain via tDCS and to obtain greater learning gain in the assembly task across online and offline sessions. A previous study [30] reported the effect of anodal tDCS for motor tasks to be nearly 3 times higher than with sham tDCS. Accordingly, our results can be considered valid. This linear relationship, however, may be characteristic of the selected tasks, and needs further validation.

In addition, normalized Z-scores were calculated to elucidate whether tDCS elicited a larger learning gain in tasks with a high cognitive demand. Larger learning gain in the active tDCS group was observed in the assembly task compared to that in the simple peg task at both online and offline sessions, as hypothesized. Successful completion of the assembly task requires higher cognitive demand, action planning [3, 13], and intentional motor control [68] to process the predetermined sequence information, which involves the DLPFC. Several studies evaluating the effectiveness of anodal tDCS on the DLPFC [69, 70] clearly separated the cognitive and motor aspects by adding a cognitive task (Stroop test and Serial-7, respectively) to the dexterity task, and reported improvement of cognitive performance. Our results are consistent with this, indicating the effectiveness of tDCS over the left DLPFC on cognitive aspects. However, the difference between our study and these previous reports is that we focused solely on the cognitive processing required to complete learning of a complex motor sequence, such as integration of independent movements and multi-element sequences that can be performed quickly and accurately [38, 39], and did not impose any explicit cognitive task on our participants. Additionally, our findings support those of previous studies reporting that the improvements are preliminarily dominated by learning the sequential characteristics of the movements, which require high cognitive load compared to motor control components [44, 47]. Therefore, our results can suggest that the effectiveness of tDCS over the left DLPFC was dependent on the cognitive processes of motor learning. Most importantly, our findings provide new evidence contributing to fill the knowledge gap on the cognitive processes of motor learning in the DLPFC and highlight the importance of the DLPFC for further motor learning in upper extremities.

In this study, there was no significant relationship between baseline performance and tDCS-induced learning gain in either of the two tasks. Previous studies [36] evaluating the effect of tDCS on left DLPFC function support our results regarding the disconnection between baseline performance and tDCS effects. This suggests that tDCS over the left DLPFC is effective in enhancing early-phase motor learning regardless of individual differences in baseline performance. Therefore, single-session tDCS on the left DLPFC may be a useful neuromodulation technique for enhancing upper-limb motor learning without specific feedback and guidance. Moreover, regarding clinical relevance, these results suggest that tDCS to the left DLPFC for upper-limb motor learning with the non-dominant hand will contribute to effective rehabilitation for individuals obliged to change their handedness due to stroke, traumatic brain injury, upper-limb trauma, or amputation.

## Limitations

There are some limitations to this study. First, only single-session tDCS in the initial stage of upper-limb motor learning was evaluated. The tDCS-induced gain in motor learning of the non-dominant hand should be evaluated in long-term continuous training, rather than in a single efficacy test. Moreover, retention of acquired skills should be evaluated. This could further enhance clinical application of tDCS to individuals obliged to change their handedness. Second, the learning effect due to repetition was shown in two PPT tasks. This is an unavoidable problem in the early phase of motor learning when rapid performance gains were obtained. Nevertheless, we found the effectiveness of tDCS beyond learning effects in the task with high cognitive demand, although the potential tDCS effects may have been underestimated. Third, physiological evidence underlying the behavioral data is lacking. Functional neuroimaging can provide more robust evidence and better understanding of the impact of tDCS on motor learning. Finally, precise electrode placement based on each individual’s brain structure could not be performed. A recent study evaluating the precision of electrode placement in electroencephalography based on the international 10/20 system referring to structural magnetic resonance imaging (MRI) data revealed the variation in electrode location in Montreal Neurological Institute coordinates [71]. The use of structural MRI in combination with neuronavigation systems might provide a more precise electrode placement, allowing for spatially-consistent current stimulation. Moreover, functional MRI can precisely measure task-related brain activation, which makes it ideal for electrode placement.

## Conclusions

This study demonstrated that tDCS significantly improved early-phase manual dexterity skill acquisition regardless of baseline performance level, particularly in tasks requiring high cognitive demand. tDCS on the left DLPFC can potentially accelerate early-phase manual dexterity skill acquisition and contribute to further understanding of the underlying neurophysiological mechanisms in the left DLPFC during this process.

## Supplementary Information

**Additional file 1**: **Table S1**. Results of 2 × 2 × 2 mixed-design analysis of variance for normalized Z scores.

## Data Availability

The datasets used and/or analyzed during the current study are available from the corresponding author on reasonable request.

## Abbreviations

DLPFC: Dorsolateral prefrontal cortex
EHI: Edinburgh Handedness Inventory
MRI: Magnetic resonance imaging
PFC: Prefrontal cortex
PPT: Purdue Pegboard Test
tDCS: Transcranial direct current stimulation
BOLD: Blood oxygenated level-dependent activity
rCBF: Regional cerebral blood flow
