# Supplementary material for: Transcranial direct current stimulation to the left dorsolateral prefrontal cortex enhances early dexterity skills with the left non-dominant hand: a randomized controlled trial
Source: J Transl Med. 2023 Feb 24;21:143. doi: 10.1186/s12967-023-03989-9 (PMC9951449; doi:10.1186/s12967-023-03989-9)
Supplement: Supplementary file 1 — Additional file 1: Table S1. Results of 2 × 2 × 2 mixed-design analysis of variance for normalized Z scores. [file 12967_2023_3989_MOESM1_ESM.docx]

|  | Degree of freedom | F value | P value | *η^2^_p_* |
| --- | --- | --- | --- | --- |
| Time  Task  Group  Time × Task  Time × Group  Task × Group  Time × Task × Group | 1  1  1  1  1  1  1 | 60.59  9.07  7.97  0.84  8.25  8.55  0.44 | 0.000  0.004  0.006  0.773  0.006  0.005  0.509 | 0.486  0.124  0.111  0.001  0.114  0.118  0.007 |

**Additional File 1. Table 1.** Results of 2 × 2 × 2 mixed-design analysis of variance for normalized Z scores.

*η^2^_p_*, partial eta squared

The group is the between-participant factor; time and task type are the within-participant factors.
